# Metronomic chemotherapy using oral cyclophosphamide and bevacizumab for recurrent cervical cancer: A multi-institutional retrospective study

**DOI:** 10.1016/j.gore.2022.101013

**Published:** 2022-05-28

**Authors:** Roze Isono-Taniguchi, Mayako Goto, Yumi Takimoto, Tomoko Ueda, Yu Wakimoto, Kayo Inoue, Kensuke Hori, Kimihiko Ito, Hiroshi Tsubamoto

**Affiliations:** a Department of Obstetrics and Gynecology, School of Medicine, Hyogo Medical University, Hyogo 663-8501, Japan; b Department of Obstetrics and Gynecology, Kansai Rosai Hospital, Hyogo, 660-8511, Japan

**Keywords:** Metronomic chemotherapy, Cyclophosphamide, Bevacizumab, Cervical cancer

## Abstract

No standard chemotherapy is available after disease progression or anaphylaxis during platinum chemotherapy among patients with recurrent cervical cancer. Here we report the efficacy and toxicities of metronomic chemotherapy consisting of 50 mg of oral cyclophosphamide (CPA) daily and intravenous 15 mg/kg of bevacizumab (BEV) repeated every 3 weeks (CPA-BEV). Treated patients were retrospectively reviewed. Adverse events and response rates were recorded according to the Common Toxicity Criteria for Adverse Events (CTCAE) ver 5.0 and Response Evaluation Criteria In Solid Tumors ver 1.1, respectively. Eleven patients had been treated with CPA-BEV between 2016 and 2021.The pathologic types were squamous cell carcinoma in seven patients, adenocarcinoma in three, and large cell neuroendocrine carcinoma in one. Nine patients had primary concurrent chemoradiotherapy (CCRT). Five patients received more than one prior chemotherapy (excluding CCRT). Six patients had progressive disease during prior platinum-based chemotherapy, four patients recurred within 6 months of the last platinum administration, and one patient had platinum anaphylaxis. Grade 3 or more hematologic toxicities and grade 2 or more non-hematological toxicities were observed in one with grade 3 neutropenia and in one with grade 2 proteinuria, respectively. The median duration of chemotherapy was 2.8 months (range 0.2–30.6 months). One patient had CR but none had PR. Median progression-free survival was 2.8 months (95 %CI: 2.1–10.7 months), and median overall survival was 13.6 months (95 %CI: 8.4–33.7 months). In conclusion, the CPA-BEV regimen showed favorable antitumor activity with minimal toxicity and is promising candidate for second-line chemotherapy.

## Highlights

• No standard chemotherapy exists for recurrent cervical cancer patients who respond poorly to platinum administration.
• Metronomic chemotherapy is a low-toxicity treatment and can maintain quality of life.
• Low-dose oral cyclophosphamide and bevacizumab (CPA-BEV) chemotherapy shows promising results in recurrent cervical cancer.

## 1 Introduction

The standard first-line chemotherapy for recurrent cervical cancer is a combination of platinum and paclitaxel, or a triple regimen of these reagents combined with bevacizumab (Tewari et al., 2017). However, if this first-line treatment fails, there is no standard treatment for second-line chemotherapy. In particular, patients who relapse within a short period after treatment with platinum-based agents do not respond well to re-administration of platinum-based agents, with relapse occurring within 12 months according to Moore's criteria (Tewari et al., 2015) and less than 7 months according to Takekuma et al. (2017). The objective of second-line chemotherapy is to palliate symptoms and improve quality of life. In the GOG240 study, only 20% of patients received post-treatment chemotherapy, and the prognosis after relapse in post-treatment patients was not reported.

Metronomic chemotherapy is a low-toxicity treatment that is expected to inhibit angiogenesis and normalize vascular architecture through frequent small-dose administration of cytotoxic anticancer agents (Mpekris et al., 2017). Bevacizumab (an anti-vascular endothelial growth factor antibody), which has similar effects, has shown high synergy when combined with weekly paclitaxel in platinum-resistant recurrent ovarian cancer (Pujade-Lauraine et al., 2014). Weekly paclitaxel administration is also included in metronomic chemotherapy. Combination chemotherapy of low-dose oral cyclophosphamide with intravenous bevacizumab (CPA-BEV) has been reported in several breast and ovarian cancers with minimal side effects (Simsek et al., 2019). In this study, we report a multicenter retrospective review of CPA-BEV treatment in patients with recurrent cervical cancer who had a platinum-resistant recurrence or a history of platinum anaphylaxis. We previously reported 4 cases with cervical cancer who had CPA-BEV, (Isono-Nakata et al., 2018) two of which were included in this report. Among other two excluded cases, one case was treated after platinum sensitive recurrence and another case was treated with CPA-BEV in two lines in between.

## 2 Materials and methods

Recurrent cervical cancer patients were enrolled in the study if their disease course progressed within 6 months after the last administration of platinum or if they had a history of platinum anaphylaxis, and subsequently received CPA-BEV between December 2016 and December 2021. The CPA-BEV treatment regimen consisted of a daily dose of 50 mg cyclophosphamide administered orally, concurrent with 15 mg/kg doses of bevacizumab administered intravenously every 3 weeks. Adverse events were evaluated according to the National Cancer Institute Common Toxicity Criteria for Adverse Events (CTCAE), version 5.0. (United States Department of Health and Human Services, 2009) Efficacy was evaluated according to the Response Evaluation Criteria In Solid Tumors (RECIST), ver. 1.1. (Eisenhauer et al., 2009). Progression-free survival (PFS) was defined as the time from the date of the first administration of oral cyclophosphamide to the date of objectively determined disease progression. In principle, computed tomography or magnetic resonance imaging was performed when progression was expected, such as worsening of symptoms or increase in tumor markers, or as a routine examination every 2–6 months. In addition, obvious worsening of symptoms was also considered as progression. Overall survival (OS) was defined as the time from the date of the first administration of oral cyclophosphamide to death. Statistical analyses were performed on the observed distributions of PFS and OS using the Kaplan–Meier method.Statistical analyses were conducted using XLSTAT (Addinsoft, Paris, France).

## 3 Results

**Fig. 1:**
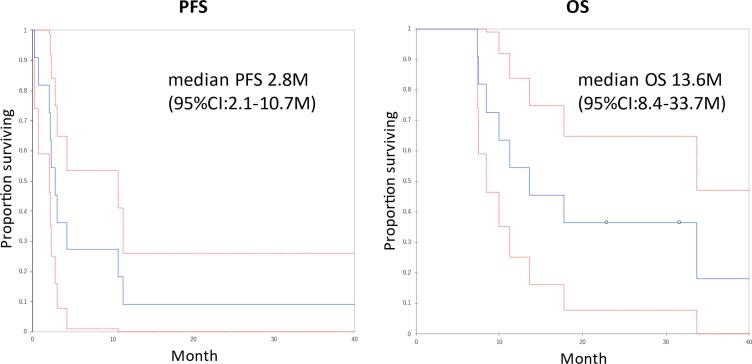
**Prognosis after CPA-BEV treatment.** The median progression-free survival (PFS) and overall survival (OS) after the administration of oral cyclophosphamide and bevacizumab for the patients with recurrent cervical cancer. Median PFS was 2.8 months (95% CI: 2.1–10.7 months) and median OS was 13.6 months (95% CI: 8.4–33.7 months). The PFS rate at 6 months were 27% (95% CI: 1.0–54%) and the OS rate at 18 months was 36% (95% CI: 7.9–64.8%).

Eight and 3 patients in Hyogo Hyogo Medical University and Kansai Rosai Hosipital were treated by multiple physicians and enrolled in this study, respectively. The median age was 50 years (range 29–76 years). The types of cervical cancer pathology represented in the study were squamous cell carcinoma in seven patients, adenocarcinoma in three, and large cell neuroendocrine carcinoma in one. Six patients received CPA-BEV because of disease progression during prior platinum-based chemotherapy, four because of disease progression within 6 months of the last platinum administration, and one because of platinum anaphylaxis. Eight patients had received concurrent chemoradiotherapy (CCRT) as prior therapy, and five patients had received two or more chemotherapy regimens (excluding CCRT). Only one patient had received bevacizumab as prior therapy (Case 4 in Table 1). If a patient was determined to have progressive disease based on RECIST criteria, the CPA-BEV regimen was continued if there was no worsening of symptoms, side effects were minimal, and the attending physician judged that the patient's progress was slow. Adverse events included grade 3 neutropenia in one patient, grade 2 proteinuria in one, grade 1 anorexia in one, and grade 1 gastrointestinal hemorrhage in one (Table 1). No non-hematologic toxicities of grade 2 or higher were observed. The median duration of chemotherapy was 2.8 months (range 0.2–30.6 months); one patient had a complete response but no patient had a partial response. Median PFS was 2.8 months (95% CI: 2.1–10.7 months) and median OS was 13.6 months (95% CI: 8.4–33.7 months). (Fig. 1) The PFS rate at 6 months was 27% (95% CI: 1.0–54%), and the OS rate at 18 months was 36% (95% CI: 7.9–64.8%).

**Table 1:** Characteristics of 11 cases.

	Prior treatment					CPA-BEV					
	FIGO	Histological type	CCRT	Chemotherapy	Indications	Adverse Events	PFS	Duration of administration	OS	Status	Subsequent therapy
				(lines)			(month)	(month)	(month)		
1	IB1	SCC	1	2	P anaphylaxis	G3 neutropenia	11.3	30.6	33.7	DOD	BSC
2	IB2	SCC	1	2	P refractory	–	40.2	24.3	40.2	NED	RT
3	IB2	Adeno	1	1	P refractory	–	4.3	4.3	8.4	DOD	Chemo*1, BSC
4	IB1	LCNEC	0	2	P refractory	–	2.2	5.8	13.6	DOD	Chemo*2
5	IVB	SCC	1	0	P refractory	G1 nausea	0.2	0.2	11.5	AWD	RT, BSC
6	IIA	Adeno	1	1	P refractory	G2 proteinuria	0.8	0.8	2.8	AWD	BSC
7	IB1	SCC	1	5	P refractory	–	2.1	2.1	7.5	DOD	BSC
8	IVB	SCC	1	1	P refractory	–	10.7	2.2	28.9	AWD	BSC
9	IVB	Adeno	0	1	P refractory	–	2.8	2.8	5.6	AWD	BSC
10	IIIB	SCC	1	0	P refractory	G1 lower gastrointestinal hemorrhage	3.0	3	7.3	DOD	RT, BSC
11	IIB	SCC	1	2	P refractory	–	2.3	2.3	9.3	DOD	BSC

FIGO, International Federation of Gynecology and Obstetrics; CCRT, Concurrent chemoradiotherapy; SCC, squamous cell carcinoma; adeno, adenocarcinoma; LCNEC, large cell neuroendocrine carcinoma; P, platinum; PFS, progression-free survival; OS, overall survival; N/A, not available; AWD, alive with disease; NED, no evidence of disease; DOD, dead of disease; BSC, best supportive care; RT, radiotherapy.

*1 Chemo: Subsequent regimens were paclitaxel, carboplatin plus bevacizumab.

*2 Chemo: Subsequent regimens were irinotecan plus cisplatin and gemcitabine, cisplatin plus bevacizumab.

## 4 Discussion

The median PFS and OS were 2.8 months and 13.6 months, respectively, of recurrent cervical cancer patients who received CPA-BEV as their second-line chemotherapy. The PFS rate at 6 months was 27% (95% CI: 1.0–54%). These outcomes compare favorably to the results of previous second-line chemotherapy studies. First, in a GOG phase II study of single agent bevacizumab for patients with recurrent cervical cancer, the primary endpoint measures of PFS for at least 6 months, median PFS, and median OS were 23.9% (90% CI, 14%–37%), 3.4 months (95% CI: 2.5–4.5 months), and 7.3 months (95% CI: 6.1–10.4 months), respectively (Monk et al., 2009). Secondly, the immune checkpoint inhibitor cemiplimab and an investigator's choice of a single cytotoxic anticancer agent were compared for second-line treatment in the GOG-3016 randomized trial (Tewari et al., 2022). The results for cemiplimab showed a median PFS of 2.8 months (95 %CI, 2.6–3.9 months) and a median OS of 12 months (95% CI: 10.3–13.5 months), but those of the cytotoxic anticancer agent showed a PFS of 2.9 months (95% CI: 2.7–3.4 months) and an OS of 8.5 months (95% CI: 7.5–9.6 months). In the GOG-3016 study, the OS at a median follow-up of 18 months for patients treated with cemiplimab was similar to that revealed in our study. Moreover, CPA-BEV treatment appears to be well-tolerated. The only grade 3 or higher adverse event in our study was neutropenia, occurring in one patient. By contrast, in the above randomized controlled trials, grade 3 or higher adverse events occurred in 45% and 53.4% of patients in the cemiplimab and anticancer chemotherapy groups, respectively. This suggests that CPA-BEV is an effective and less toxic treatment for recurrent cervical cancer in the second-line and beyond, compared with not only single-agent anticancer chemotherapy but also immune checkpoint agents.

In addition to inhibiting angiogenesis, CPA-BEV is believed to enhance tumor immunity by optimizing vascularization within the tumor microenvironment and modulating regulatory T cells (Simsek et al., 2019). Although immune checkpoint agents for cervical cancer have not been approved in Japan, pembrolizumab has recently been approved for solid tumors presenting with high tumor mutation burden (TMB-H, ≥10 Muts/Mb). TMB-H has been reported in 21% of cervical cancer cases, and the response rate of cervical cancer to immune checkpoint inhibitor treatment is as high as 31% (Marabelle et al., 2020). Before the results of GOG-3016, the usefulness of 1st line pembrolizumab was also reported, and expectations for immune-mediated drug therapy for the treatment of cervical cancer are high (Colombo et al., 2021). Although the present study could not investigate CPA-BEV therapy and its immunostimulatory effects in cervical cancer, a phase II trial of CPA-BEV plus pembrolizumab has been reported in recurrent ovarian cancer, (Zsiros et al., 2021) and there is potential for future development of combination therapy with CPA-BEV and immune checkpoint agents in cervical cancer as well. The limitation of this study is that only one patient who received prior bevacizumab treatment was enrolled in this study. The concept of ‘bevacizumab beyond bevacizumab’ was established after randomized control study of platinum sensitive recurrent ovarian cancer (Pignata, Lancet 2021), however, it remains obscure for cervical cancer.

## 5 Conclusion

The CPA-BEV regimen appears to be a promising treatment for recurrent cervical cancer with minimal toxicity and antitumor effects.

## Consent

Written informed consent was obtained from all patients for publication of the case series.

## Funding

No external funding was received.

### CRediT authorship contribution statement

**Roze Isono-Taniguchi:** Conceptualization, Methodology, Investigation, Writing – original draft. **Mayako Goto:** Investigation, Project administration, Writing – review & editing. **Yumi Takimoto:** Investigation, Methodology, Supervision, Project administration, Writing – review & editing. **Tomoko Ueda:** Investigation, Project administration, Writing – review & editing. **Yu Wakimoto:** Investigation, Project administration, Writing – review & editing. **Kayo Inoue:** Investigation, Project administration, Writing – review & editing. **Kensuke Hori:** Investigation, Project administration, Writing – review & editing. **Kimihiko Ito:** Investigation, Project administration, Supervision. **Hiroshi Tsubamoto:** .

## Declaration of Competing Interest

The authors declare that they have no known competing financial interests or personal relationships that could have appeared to influence the work reported in this paper.
